# Global tuberculosis challenges.

**DOI:** 10.3201/eid0403.980316

**Published:** 1998

**Authors:** K G Castro

**Affiliations:** Centers for Disease Control and Prevention, Atlanta, Georgia, USA.


### Global Tuberculosis Challenges

**Kenneth G. Castro**
Centers for Disease Control and Prevention, Atlanta, Georgia, USA

Mario Raviglione, World Health Organization (WHO), described the epidemiology of global tuberculosis (TB) using surveillance data available to WHO from 212 countries and data from a recent survey of antituberculosis drug resistance in 32 countries. Countries were categorized according to the degree of TB directly observed treatment strategy (DOTS) implementation. Performance of national TB programs was assessed by using treatment outcome indicators. In 1996, 3.8 million TB cases (887,731 from areas with DOTS) were reported to WHO. In developing countries, the bulk of TB cases are found in all age groups of native-born populations, while in many industrialized countries a large proportion of TB cases are in foreign-born residents. In countries of the former Soviet Union, TB cases and deaths have doubled in just a few years. Drug resistance and HIV infection related to TB are found only in limited foci. Acquired multidrug-resistant TB (MDRTB) was present in 27% to 54% of culture-positive TB cases from the Baltic countries and Russia. The effect of the HIV epidemic on TB has been major in Africa, where HIV seroprevalence among TB cases is 50% to 70% and TB case notifications have at times tripled. Countries with inadequate TB control are particularly exposed to the consequences of both epidemics. In Southeast Asia, cases are increasing, and MDRTB is common in Thailand, China, and Vietnam.

One hundred eighty-one countries and territories (97% of the global population) have reported on the status of DOTS to WHO. Of these, 96 implemented DOTS (63 countrywide). Approximately 32% of the global population lives in areas where DOTS is available. Twenty countries have adopted DOTS since the 1996 survey, and an additional 9% of the global population were benefitting from it. However, most of these new countries had small populations; DOTS was only slowly implemented in countries with high TB prevalence. In areas that used DOTS, treatment outcome evaluation remains high (94%), and treatment success rose from 76% in 1994 to 78% in 1995. In areas that did not use DOTS, 45% of reported TB cases were not evaluated, and treatment success remained low (45%). Among the 22 countries with the highest TB prevalence, six showed progress in DOTS implementation, seven showed little progress, and nine did not implement DOTS. In summary, TB remains an important public health problem in many areas of the world where DOTS has not been implemented. Because treatment outcomes were better in countries where DOTS has been used, the strategy needs to be expanded rapidly and new tools to facilitate its implementation need to be developed.

Barry Bloom, Howard Hughes Medical Institute, described advances in TB vaccine development. The available bacillus Calmette-Guerin (BCG) vaccine has a demonstrated efficacy ranging from no protection to 80% protection. Most recently, a meta analysis estimated that the overall efficacy of BCG is 50%. Because of case reports of disseminated BCG infection, this vaccine is contraindicated in immunocompromised persons, and safer and more efficacious vaccines are clearly needed. Identifying such new vaccines for use in humans will take several years. However, recent advances in this area provide optimism. Recent research activities have improved our understanding of the immunologic response to *Mycobacterium tuberculosis* and identified major protein antigens of *M. tuberculosis* and recombinant BCG forms that overexpress protective antigens. Additionally, avirulent auxotrophic mutants of both BCG and *M. tuberculosis* have been used in animal models. The recent sequencing of the *M. tuberculosis* genome has presented additional opportunities to identify virulence factors that could be deleted and other target sites that could be genetically engineered. DNA constituents can also be used to develop candidate vaccines. In animal studies, subunit vaccines consisting of pooled mycobacterial culture-filtrate proteins have been protective. Auxotrophic mutants may also prove useful in immunocompromised patients, as may recombinant BCG vaccines that secrete host-specific cytokines. Clearly, a major national effort is required for TB vaccine development, recommendations on policies and priorities, and cooperation between the government and private sector in these efforts.

Christopher Murray, Harvard School of Public Health, described a mathematical model developed to forecast the future impact of improvements in TB prevention and control. Specifically, this model projected the number of TB cases and deaths averted through the year 2050. Different scenarios were simulated to project the effect of adding TB vaccines to existing interventions. Six specific scenarios assessed the effect of vaccines (with efficacy levels of 20%, 50%, and 80%) to protect from *M. tuberculosis* infection, as well as the effect of vaccines of the same levels of efficacy to protect latently infected persons from “breakdown” to active TB. Although a TB infection vaccine with 20% efficacy would prevent more than 30 million TB cases, the best protection is obtained from a TB breakdown vaccine with 80% efficacy, which would prevent almost 130 million TB cases. The breakdown vaccine could be used in the large number of persons with latent *M. tuberculosis* infection, now estimated at almost one third of the world’s population. Such anticipated gains justify the effort to develop better TB vaccines.

Denise Garrett, Centers for Disease Control and Prevention, presented the findings of recent tuberculin skin test studies regarding the risk for TB among health-care workers in Thailand and Brazil. In Thailand, 35% of 911 health-care workers had a positive test at the 15-mm cutoff, while 69% were positive at the 10-mm cutoff. BCG scar was associated with positive skin test reaction at the lower cutoff value, but not at the 15-mm cutoff. Additionally, tuberculin skin test reactivity correlated with more than 1 year’s employment as a health-care worker, and with occasional or frequent patient contact. In Brazil, 48% of 524 health-care workers had a reaction of 10 mm, while 26% had a reaction of 15 mm. As in Thailand, BCG scar correlated only with 10-mm skin test reactivity but not with 15-mm. Workers with occasional or frequent patient contact were also more likely to have a positive tuberculin test. Factors that appear to contribute to the risk for TB in these workers include delays in the diagnosis of TB, inadequate isolation practices, and lack of personal protection during high-risk procedures. Important measures to reduce the risk for TB in these settings include increasing the awareness and training of health-care workers about the risk for TB, improving the ability to establish the diagnosis of TB by smear microscopy, reducing the need for hospitalization of TB patients, considering the establishment of chest clinics at separate times or in separate areas, and improving ventilation by keeping windows open. Laboratories should contain all needed safety features.

